# Competitive level is associated with dietary practices but not sports nutrition knowledge in collegiate athletes: associations with body composition profiles

**DOI:** 10.1186/s13102-026-01974-5

**Published:** 2026-08-20

**Authors:** Mohamed Osama, Mohamed Bakr, Mahmoud Al Sayed

**Affiliations:** Department of Health Sciences Sports, Faculty of Sport Science, Benha University, Benha, Egypt

**Keywords:** Sports nutrition, Nutrition knowledge, Dietary intake, Body composition, Collegiate athletes, Competitive level, Protein intake

## Abstract

**Background:**

Sports nutrition knowledge may influence athletes’ dietary practices, but its relationship with dietary intake and body composition remains unclear, particularly across different competitive levels. This study examined the associations among sports nutrition knowledge, dietary intake, and body composition and compared these variables between amateur and elite collegiate athletes.

**Methods:**

This cross-sectional study included 100 male collegiate athletes (60 amateur and 40 elite). Sports nutrition knowledge was assessed using an adapted Sports Nutrition Knowledge Questionnaire (SNKQ) developed for this study based on the conceptual framework of the Nutrition for Sport Knowledge Questionnaire (NSKQ). Dietary intake was evaluated using three-day food records, and body composition was measured by multifrequency bioelectrical impedance analysis. Between-group differences were analyzed using independent-samples *t*-tests with 95% confidence intervals and Cohen’s *d*. Pearson correlation and multiple linear regression analyses were used to examine associations and identify predictors of body composition.

**Results:**

Elite athletes consumed significantly more energy, protein, and carbohydrates and reported higher meal frequency and daily fluid intake than amateur athletes (all *p* < 0.001). Sports nutrition knowledge scores were similar between groups (*p* = 0.57). Although elite athletes had lower body fat and higher skeletal muscle mass percentages, these differences were not statistically significant. Dietary intake, particularly protein intake, showed stronger associations with body composition than sports nutrition knowledge. Multiple regression analysis identified protein intake and competitive level as independent predictors of body composition.

**Conclusions:**

Competitive level was associated with healthier dietary practices but not with sports nutrition knowledge among collegiate athletes. Dietary intake, particularly protein intake, was more strongly associated with body composition than nutrition knowledge. These findings suggest that improving dietary behaviors may have greater practical value than increasing nutrition knowledge alone. Longitudinal and intervention studies are needed to confirm these relationships.

**Supplementary Information:**

The online version contains supplementary material available at 10.1186/s13102-026-01974-5.

## Introduction

Body composition is an important characteristic associated with athletic performance, training adaptation, and long-term health. The balance between fat mass and lean mass is related to strength, power, movement efficiency, endurance, recovery, and physiological responses to exercise [1, 2]. Because optimal body composition varies according to the specific demands of each sport, athletes aim to develop sport-specific body composition profiles while maintaining adequate energy availability and overall health.

Body composition is associated with multiple interacting factors, including training load, dietary intake, recovery practices, hormonal regulation, and environmental conditions [3, 4]. Among these, nutrition represents a modifiable factor that may contribute to body composition and athletic performance. Adequate energy availability, together with appropriate carbohydrate and protein intake, is associated with muscle protein synthesis, glycogen restoration, recovery, immune function, and adaptation to training [5, 6]. Conversely, inadequate dietary intake or chronic low energy availability has been associated with impaired recovery, reduced training quality, compromised lean tissue maintenance, and an increased risk of relative energy deficiency in sport (RED-S) [4, 6]. Current sports nutrition guidelines therefore recommend individualized nutritional strategies that align with training and competition demands [7, 8].

Appropriate dietary practices are considered an important component of athlete preparation and are partly informed by sports nutrition knowledge. Sports nutrition knowledge encompasses recommendations related to energy and macronutrient intake, hydration, recovery nutrition, body composition management, and dietary supplement use [9]. However, evidence suggests that nutrition knowledge alone is not consistently associated with healthier dietary behaviors or improved physiological outcomes [9, 10]. Food choices are influenced by numerous additional factors, including food availability, financial resources, time constraints, coaching support, and access to qualified sports nutrition professionals [10, 11]. Consequently, athletes with comparable nutrition knowledge may demonstrate different dietary behaviors and levels of adherence to current nutritional recommendations. Recent studies have also reported widespread dietary supplement use among athletes that is not always consistent with current evidence-based recommendations, further illustrating that nutrition-related behaviors do not necessarily reflect sports nutrition knowledge alone [12].

The Nutrition for Sport Knowledge Questionnaire (NSKQ), developed by Trakman and colleagues, provides a widely accepted framework for assessing sports nutrition knowledge in athletic populations [13]. In the present study, sports nutrition knowledge was assessed using an adapted Sports Nutrition Knowledge Questionnaire (SNKQ) developed specifically for collegiate athletes. The adapted instrument was based on the conceptual framework of the NSKQ while incorporating additional items relevant to the nutritional practices, dietary behaviors, and educational context of the study population.

Dietary intake represents the behavioral component through which nutrition recommendations are implemented in daily practice. Adequate protein intake is associated with muscle protein synthesis, recovery, and lean mass maintenance, whereas sufficient carbohydrate intake supports glycogen restoration and repeated high-intensity exercise [5, 14]. Nevertheless, previous studies indicate that many athletes do not consistently achieve recommended energy and macronutrient intakes despite demonstrating moderate or good sports nutrition knowledge [9, 10]. Recent intervention studies have further shown that individualized nutrition programs have reported improvements in dietary intake, body composition, and performance-related outcomes following individualized nutrition programs [15].

Competitive level may also be associated with dietary behaviors and body composition. Athletes competing at higher levels often train within more structured performance environments and may have greater access to coaching, nutrition professionals, and other performance support services than amateur athletes [3, 16]. However, body composition is also associated with long-term training exposure, recovery practices, lifestyle factors, and individual characteristics. Examining sports nutrition knowledge, dietary intake, and competitive level together may therefore provide a more comprehensive understanding of the factors associated with body composition in collegiate athletes.

Although sports nutrition knowledge, dietary intake, and body composition have each been widely investigated, relatively few studies have examined these variables simultaneously in collegiate athletes while considering differences in competitive level. Consequently, it remains unclear whether differences in competitive level are accompanied by differences in nutrition knowledge, dietary behaviors, and body composition within the same athletic population. Addressing these relationships may contribute to a more comprehensive understanding of factors associated with athlete nutrition and body composition.

Therefore, the primary aim of this study was to examine the relationships among sports nutrition knowledge, dietary intake, and body composition in collegiate athletes and to compare these variables between amateur and elite competitors. A secondary aim was to identify variables independently associated with body composition using multiple linear regression analysis. We hypothesized that elite athletes would report more favorable dietary practices than amateur athletes despite similar sports nutrition knowledge, and that dietary intake, particularly energy and protein intake, would demonstrate stronger associations with body composition than nutrition knowledge.

## Materials and methods

### Study design and participants

This cross-sectional observational study examined the relationships among sports nutrition knowledge, dietary intake, and body composition in collegiate athletes. The study was conducted at the Faculty of Sport Sciences, Benha University, Egypt, between March and April 2026 during the pre-competitive period to reduce the influence of seasonal changes in training load, dietary intake, and body composition.

Male collegiate athletes were recruited from university teams in football, basketball, volleyball, athletics, and wrestling. Eligible participants were aged 18 years or older, had completed at least one competitive season, and regularly participated in organized university competitions. Athletes were excluded if they had chronic medical or metabolic conditions, musculoskeletal injuries that limited training, or any condition likely to affect nutritional status or body composition.

A total of 100 athletes met the eligibility criteria and completed all assessments. Participants were classified as elite (*n* = 40) or amateur (*n* = 60) according to predefined competitive-level criteria. Competitive level was selected a priori as the primary grouping variable because the study aimed to compare nutritional characteristics between athletes with different competitive standards. Although participants represented multiple sport disciplines, the sample size within each sport was insufficient to support adequately powered sport-specific analyses; therefore, all statistical analyses were conducted according to competitive level.

**Fig. 1:**
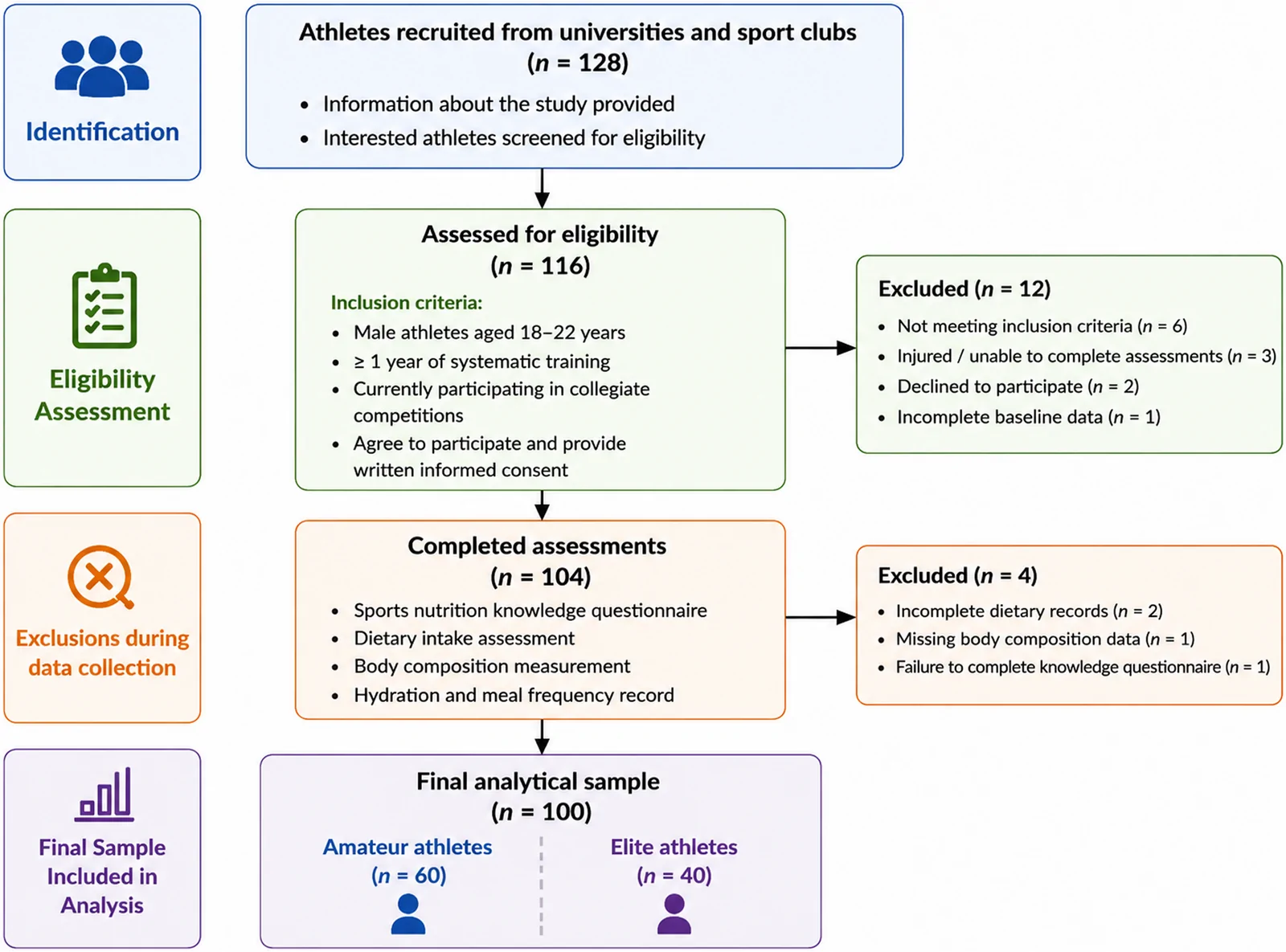
Participant flow diagram. Overview of participant recruitment, eligibility assessment, exclusions, completion of study assessments, and allocation to the final analytical sample of amateur (*n* = 60) and elite (*n* = 40) collegiate athletes

The participant recruitment process, eligibility assessment, exclusions, and final analytical sample are presented in Fig. 1.

### Sample size calculation

Sample size was determined a priori using a correlation-based approach because the primary objective was to examine associations among sports nutrition knowledge, dietary intake, and body composition. Assuming an expected correlation coefficient of *r* = 0.30, a two-sided significance level of α = 0.05, and 80% statistical power, the minimum required sample size was 85 participants. To account for potential missing data and incomplete dietary records, the recruitment target was increased to 100 athletes, providing adequate statistical power for all planned analyses.

### Classification of competitive level

Because no universally accepted definition of **elite** and **amateur** athletes exists across different sports, participants were classified using study-specific operational criteria informed by published athlete classification frameworks [17, 18]. These frameworks recommend transparent reporting of athlete characteristics using objective indicators of competitive standard and training history rather than relying solely on self-reported competitive status.

Competitive level was defined before participant recruitment and applied consistently throughout the study to ensure objective group allocation.

Athletes were classified as elite (*n* = 40) if they:

- were members of officially registered university or sports club teams;
- had regularly competed in regional, national, or inter-university championships during the previous competitive season;
- completed ≥ 5 coach-supervised training sessions per week; and.
- had accumulated ≥ 3 years of competitive experience.

Athletes were classified as amateur (*n* = 60) if they participated in organized university or recreational sports but did not meet all of the elite classification criteria. Typically, these athletes trained fewer than five coach-supervised sessions per week, competed primarily at recreational or university level, and had less competitive experience than athletes classified as elite.

These operational criteria were developed specifically for the present study to distinguish athletes with sustained competitive participation and structured training within a collegiate population. Accordingly, they should be interpreted as study-specific operational definitions rather than standardized athlete classification criteria applicable across different sports or research settings.

### Demographic and training characteristics

Demographic and training data were collected using a standardized questionnaire before study assessments. Recorded variables included age, height, body mass, primary sport, years of competitive experience, weekly training frequency, and average training duration per session. Training frequency and competitive experience were collected to characterize the study sample and to verify participant eligibility according to the predefined competitive-level classification criteria. No information was collected regarding previous nutrition education, access to sports nutrition professionals, socioeconomic status, or food availability because these variables were beyond the scope of the present study.

Because all participants were male, sex was not included as a covariate in the analyses. These variables were used to describe the study population and characterize the amateur and elite athlete groups.

### Body composition assessment

Body composition was assessed using a multifrequency segmental bioelectrical impedance analysis system (InBody 770, InBody Co., Ltd., Seoul, Republic of Korea). The device measures tissue impedance at six frequencies (1, 5, 50, 250, 550, and 1000 kHz) to estimate body composition across different body segments.

Multifrequency BIA was selected because it provides a rapid, non-invasive, and practical method for assessing body composition in athletic populations. Although dual-energy X-ray absorptiometry (DXA) is considered the reference method for body composition assessment, multifrequency BIA provides a practical and validated alternative for athlete monitoring under standardized testing conditions [2, 19, 20].

All assessments were performed between 07:00 and 10:00 h under standardized laboratory conditions by the same trained investigators. Participants were instructed to avoid vigorous exercise for at least 12 h, refrain from eating or consuming caloric beverages for at least 3 h before testing, maintain their usual hydration status, empty their bladder immediately before assessment, and remove all metallic accessories. Measurements were obtained with participants standing barefoot while holding the hand electrodes according to the manufacturer’s instructions. Each assessment required approximately 60 s.

The following variables were recorded: body fat percentage (%), skeletal muscle mass percentage (%), body mass (kg), and body mass index (BMI, kg/m²).

### Sports nutrition knowledge assessment

Sports nutrition knowledge was assessed using an adapted Sports Nutrition Knowledge Questionnaire (SNKQ) developed specifically for this study. The questionnaire was based on the conceptual framework and core domains of the Nutrition for Sport Knowledge Questionnaire (NSKQ) described by Trakman et al. [13] and expanded to better reflect the nutritional practices and educational needs of collegiate athletes.

The adapted SNKQ comprised 120 items across four domains: food choices and nutrient sources, weight management, nutritional habits and behaviors, and sport-specific nutrition. Additional items addressing meal planning, hydration, dietary practices, and supplement knowledge were developed from current sports nutrition guidelines and recent literature to improve the relevance of the instrument.

The questionnaire was prepared in Arabic and reviewed by experts in sports nutrition, exercise physiology, and sport sciences to evaluate content relevance, clarity, and comprehensiveness. Revisions were made following expert feedback to improve content validity. The adapted questionnaire demonstrated good internal consistency in the present sample (Cronbach’s α = 0.84), supporting its reliability for assessing sports nutrition knowledge in collegiate athletes.

Participants completed the questionnaire under supervised conditions without access to external resources or discussion with other participants. Each correct response was awarded one point, while incorrect or unanswered responses received zero points. Item scores were summed to generate domain-specific and total sports nutrition knowledge scores, with higher scores indicating greater nutrition knowledge.

### Dietary intake assessment

Habitual dietary intake was assessed using a prospective 3-day food record, including two training weekdays and one weekend day to capture usual dietary intake while accounting for day-to-day variation. Participants were instructed to maintain their normal eating habits and record all foods, beverages, and dietary supplements as soon as possible after consumption to minimize recall bias.

Before data collection, trained investigators provided standardized verbal and written instructions on completing the food records, including food type, portion size, cooking method, meal timing, snacks, fluid intake, and supplement use. Completed records were reviewed individually to verify completeness, clarify ambiguous entries, and confirm portion sizes before nutrient analysis.

Dietary data were analyzed using Nutritics Research Edition (Nutritics Ltd., Dublin, Ireland). The following variables were calculated:

- Total energy intake (kcal/day)
- Relative energy intake (kcal/kg/day)
- Protein intake (g/kg/day)
- Carbohydrate intake (g/kg/day)
- Fat intake (% of total energy intake)
- Reported fluid intake (L/day)
- Meal frequency (meals/day)

The 3-day food record is widely accepted as a practical method for estimating habitual dietary intake in athletic populations while balancing participant burden and assessment accuracy.

### Data collection procedures

Data were collected over three visits within a two-week period using standardized protocols.

#### Visit 1: baseline assessment

Participants provided written informed consent before completing a standardized questionnaire on demographic characteristics, primary sport, competitive experience, training frequency, and training duration. Standing height, body mass, and body composition were then measured.

#### Visit 2: body composition assessment

Body composition was assessed under standardized morning laboratory conditions using the InBody 770 multifrequency bioelectrical impedance analyzer. All measurements were performed by the same trained investigators to minimize measurement variability.

#### Visit 3: nutrition assessment

Participants completed the adapted Sports Nutrition Knowledge Questionnaire (SNKQ) under researcher supervision and submitted their completed 3-day food records. Dietary records were reviewed for completeness and consistency, and any unclear or missing information was verified directly with participants before nutrient analysis using Nutritics Research Edition.

**Fig. 2:**
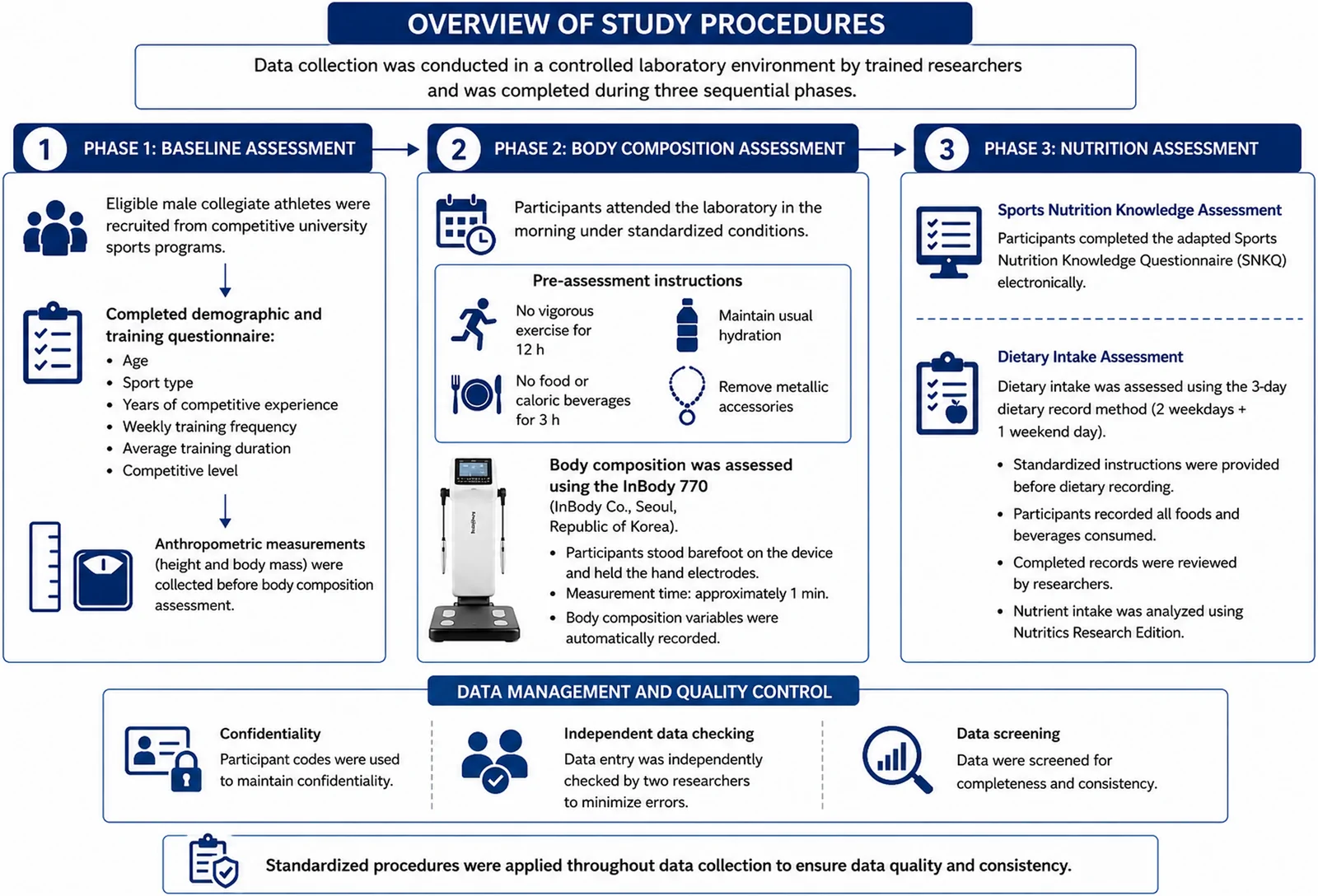
Overview of the study procedures. Participants completed three sequential phases consisting of baseline assessment, body composition assessment, and nutrition assessment. Standardized procedures were applied throughout data collection to ensure consistency and data quality. BIA, bioelectrical impedance analysis; SNKQ, Sports Nutrition Knowledge Questionnaire

All assessments were performed by the same research team following standardized procedures to ensure consistency and reduce measurement error. An overview of participant recruitment, assessment procedures, and quality-control measures is presented in Fig. 2.

### Statistical analysis

Statistical analyses were performed using IBM SPSS Statistics version 29.0 (IBM Corp., Armonk, NY, USA). Statistical significance was set at *p* < 0.05 for all analyses.

Data normality was assessed using the Shapiro-Wilk test, supported by visual inspection of histograms and normal Q-Q plots. Continuous variables are presented as mean ± standard deviation (SD), whereas categorical variables are presented as frequencies and percentages.

Differences between elite and amateur athletes were examined using independent-samples *t*-tests. Results are reported as mean differences with 95% confidence intervals (95% CI) and Cohen’s d effect sizes. Effect sizes were interpreted as small (0.20), moderate (0.50), and large (0.80). Because all comparisons were predefined according to the study objectives and represented distinct outcome variables, no adjustment for multiple comparisons was applied. Sport discipline was not included as a covariate because participant numbers within individual sports were insufficient to support reliable subgroup or multivariable analyses.

Associations among sports nutrition knowledge, dietary intake variables, and body composition characteristics were evaluated using Pearson’s product-moment correlation coefficients. Correlation strength was interpreted as weak (*r* = 0.10 to 0.29), moderate (*r* = 0.30 to 0.49), and strong (*r* ≥ 0.50).

To identify independent predictors of body composition, two multiple linear regression models were constructed using body fat percentage (%) and skeletal muscle mass percentage (%) as the dependent variables. Independent variables entered simultaneously into both models were sports nutrition knowledge score, relative energy intake (kcal/kg/day), protein intake (g/kg/day), carbohydrate intake (g/kg/day), and competitive level (elite vs. amateur).

Regression assumptions were evaluated before model interpretation. Multicollinearity was assessed using variance inflation factor (VIF) and tolerance statistics, with VIF < 5.0 and tolerance > 0.20 considered acceptable. Normality of residuals was examined using standardized residual histograms and normal probability plots. Linearity and homoscedasticity were evaluated by visual inspection of scatterplots of standardized predicted values against standardized residuals. Independence of residuals was assessed using the Durbin-Watson statistic, with values of 2.03 for the body fat model and 1.96 for the skeletal muscle mass model, indicating no evidence of residual autocorrelation. Overall, no meaningful violations of the assumptions for multiple linear regression were identified.

Regression results are presented as standardized regression coefficients (β), p-values, variance inflation factors (VIF), coefficients of determination (R²), adjusted R², and the overall F statistic for each model.

This study is reported in accordance with the Strengthening the Reporting of Observational Studies in Epidemiology (STROBE) statement for cross-sectional studies. A completed STROBE checklist is provided as Supplementary Material.

## Results

### Participant characteristics and dietary intake according to competitive level

**Fig. 3:**
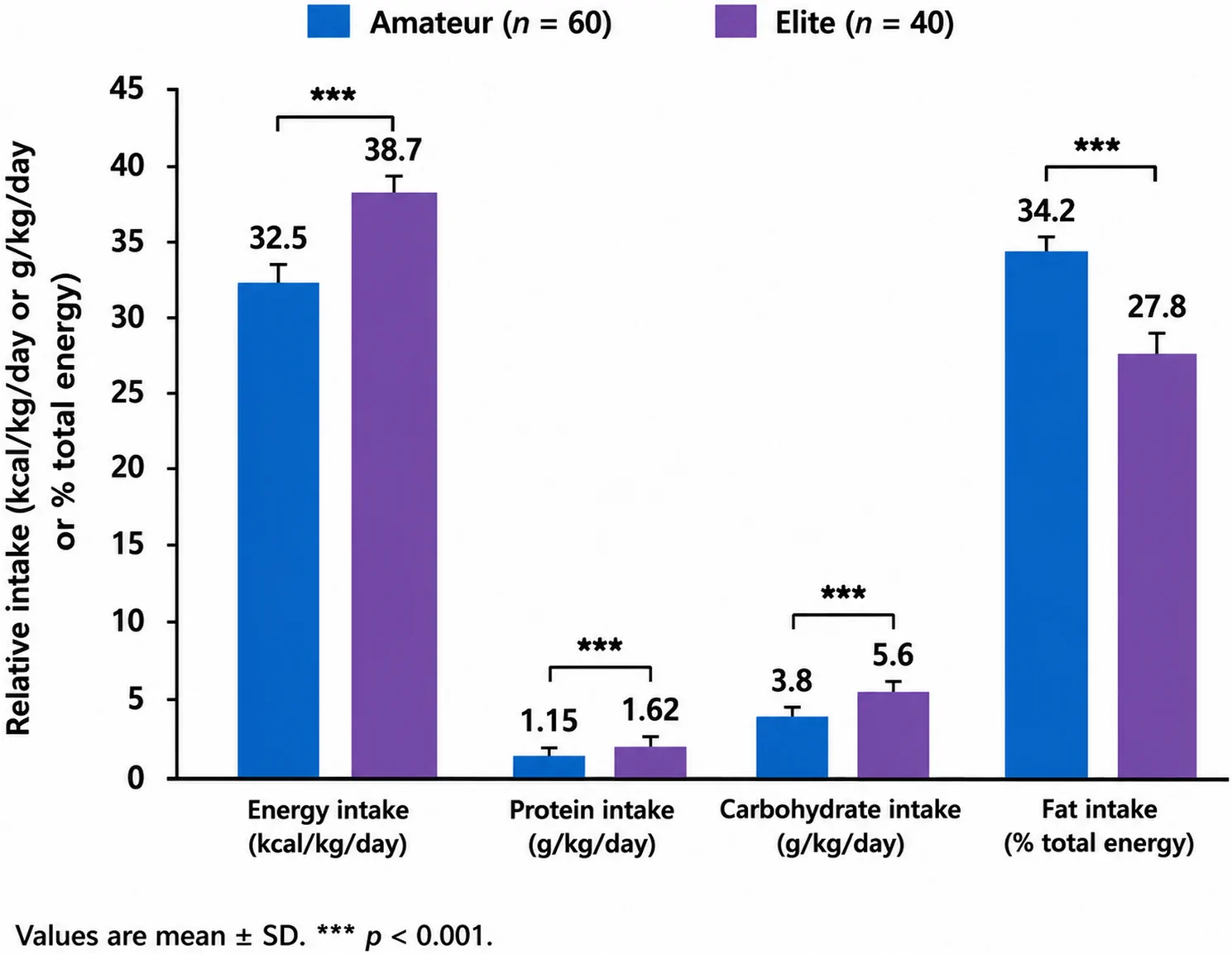
Dietary intake profiles of amateur and elite collegiate athletes. Values are presented as mean ± SD. Elite athletes consumed significantly more energy, protein, and carbohydrates, whereas amateur athletes derived a greater proportion of total energy from fat (*p < 0.001)

**Fig. 4:**
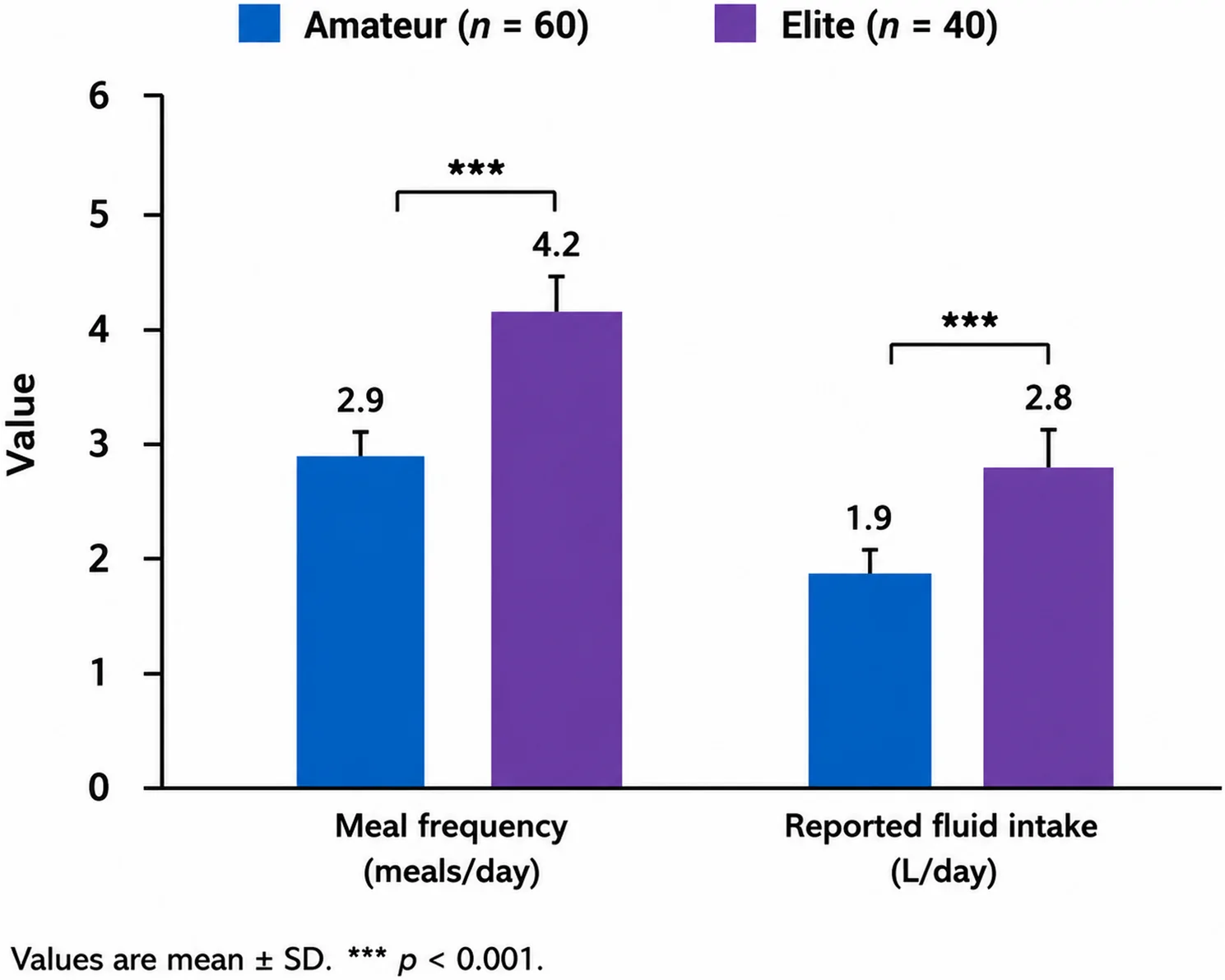
Meal frequency and reported fluid intake according to competitive level. Meal frequency (meals/day) and reported daily fluid intake (L/day) in amateur (*n* = 60) and elite (*n* = 40) collegiate athletes. Data are presented as mean ± SD. ***p < 0.001 between groups

A total of 100 male collegiate athletes participated in the study, including 60 amateur and 40 elite athletes Participants represented five sports (football, basketball, volleyball, athletics, and wrestling). Because participant numbers within individual sports were insufficient for adequately powered subgroup analyses, all results are presented according to competitive level, the primary grouping variable. Participant characteristics and dietary intake are presented in Table 1; Figs. 3 and 4.

**Table 1:** Physical characteristics and dietary intake according to competitive level

Variable	Amateur (*n* = 60)	Elite (*n* = 40)	Mean Difference (Elite, Amateur) (95% CI)	*p*-value	Cohen’s d
Age (years)	18.33 ± 0.70	18.25 ± 0.43	-0.08 (-0.31, 0.15)	0.49	0.14
Height (cm)	177.73 ± 5.22	176.16 ± 5.04	-1.57 (-3.66, 0.52)	0.14	0.30
Body mass (kg)	73.37 ± 9.98	73.58 ± 10.38	0.21 (-4.04, 4.46)	0.92	0.02
BMI (kg/m²)	23.21 ± 2.58	23.70 ± 3.00	0.49 (-0.72, 1.70)	0.42	0.18
Energy intake (kcal/kg/day)	32.50 ± 5.80	38.70 ± 6.20	6.20 (3.66, 8.74)	**< 0.001**	1.02
Protein intake (g/kg/day)	1.15 ± 0.30	1.62 ± 0.35	0.47 (0.33, 0.61)	**< 0.001**	1.45
Carbohydrate intake (g/kg/day)	3.80 ± 1.20	5.60 ± 1.50	1.80 (1.24, 2.36)	**< 0.001**	1.31
Fat intake (% of total energy)	34.20 ± 6.10	27.80 ± 5.40	-6.40 (-8.78, -4.02)	**< 0.001**	1.11
Meal frequency (meals/day)	2.90 ± 0.80	4.20 ± 0.90	1.30 (0.96, 1.64)	**< 0.001**	1.52
Daily fluid intake (L/day)	1.90 ± 0.60	2.80 ± 0.70	0.90 (0.62, 1.18)	**< 0.001**	1.38

Abbreviations: *BMI* body mass index, *CI* confidence interval, *kcal* kilocalories, *d* Cohen’s effect size

Bold values indicate statistically significant results (*p* < 0.05)

No significant differences were observed between groups for age, height, body mass, or BMI (all *p* > 0.05).

Elite athletes reported significantly greater relative energy intake (38.7 ± 6.2 vs. 32.5 ± 5.8 kcal/kg/day, *p* < 0.001), protein intake (1.62 ± 0.35 vs. 1.15 ± 0.30 g/kg/day, *p* < 0.001), carbohydrate intake (5.6 ± 1.5 vs. 3.8 ± 1.2 g/kg/day, *p* < 0.001), meal frequency (4.2 ± 0.9 vs. 2.9 ± 0.8 meals/day, *p* < 0.001), and reported daily fluid intake (2.8 ± 0.7 vs. 1.9 ± 0.6 L/day, *p* < 0.001) than amateur athletes. Conversely, amateur athletes obtained a greater proportion of daily energy from fat than elite athletes (34.2 ± 6.1% vs. 27.8 ± 5.4%, *p* < 0.001). Overall, elite athletes reported higher energy and macronutrient intake, greater meal frequency, and greater daily fluid intake than amateur athletes.

### Sports nutrition knowledge according to competitive level

**Fig. 5:**
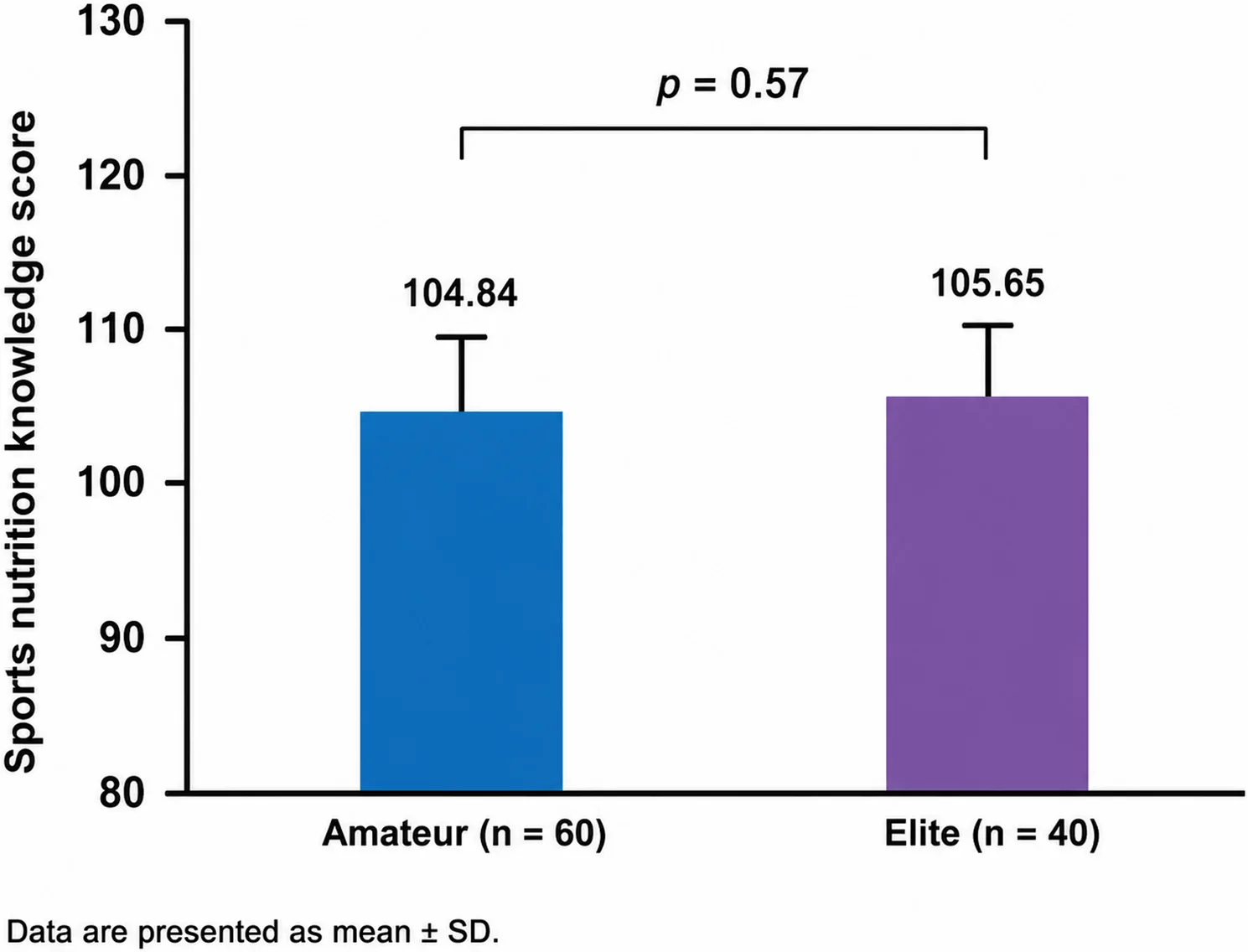
Total sports nutrition knowledge scores according to competitive level. Total sports nutrition knowledge scores in amateur (*n* = 60) and elite (*n* = 40) collegiate athletes. Data are presented as mean ± SD. No significant difference was observed between groups (*p* = 0.57)

Sports nutrition knowledge scores are presented in Table 2; Fig. 5.

**Table 2:** Sports nutrition knowledge scores according to competitive level

Variable	Amateur (*n* = 60)	Elite (*n* = 40)	Mean Difference (95% CI)	*p*-value	Cohen’s d
Food consumption	21.84 ± 1.78	22.10 ± 1.46	0.26 (-0.40, 0.92)	0.44	0.16
Weight management	16.08 ± 2.05	16.17 ± 1.73	0.09 (-0.68, 0.86)	0.82	0.05
Nutritional habits and behaviors	32.70 ± 3.53	32.90 ± 3.44	0.20 (-1.16, 1.56)	0.77	0.06
Diets for athletes	34.28 ± 3.36	34.47 ± 2.89	0.19 (-1.09, 1.47)	0.77	0.06
Total knowledge score	104.84 ± 7.10	105.65 ± 7.38	0.81 (-2.04, 3.66)	0.57	0.11

Abbreviations: *CI* confidence interval, *d* Cohen’s effect size

No significant differences were observed between amateur and elite athletes for the total sports nutrition knowledge score or any questionnaire domain (all *p* > 0.05). Although elite athletes achieved slightly higher mean scores across all domains, the differences were small and not statistically significant. The overall sports nutrition knowledge score was comparable between elite and amateur athletes (105.65 ± 7.38 vs. 104.84 ± 7.10; *p* = 0.57, Cohen’s *d* = 0.11).

### Body composition profiles according to competitive level

**Fig. 6:**
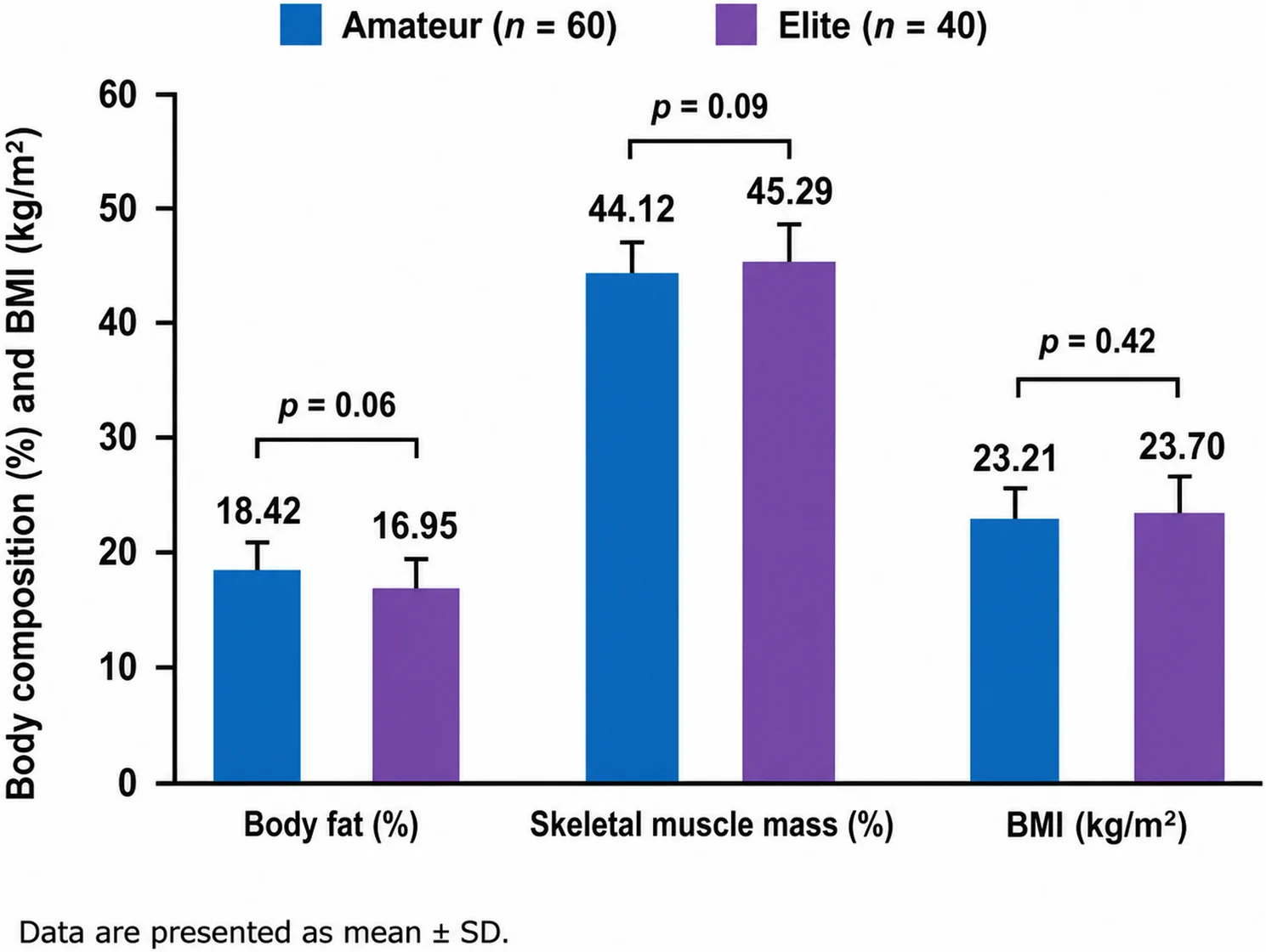
Body composition according to competitive level. Body fat percentage, skeletal muscle mass percentage, and body mass index (BMI) in amateur (*n* = 60) and elite (*n* = 40) collegiate athletes. Data are presented as mean ± SD. Between-group p-values are shown above each comparison

Body composition outcomes are presented in Table 3; Fig. 6.

**Table 3:** Body composition characteristics according to competitive level

Variable	Amateur (*n* = 60)	Elite (*n* = 40)	Mean Difference (95% CI)	*p*-value	Cohen’s d
Body fat percentage (%)	18.42 ± 3.37	16.95 ± 4.45	-1.47 (-3.01, 0.07)	0.06	0.39
Skeletal muscle mass percentage (%)	44.12 ± 3.01	45.29 ± 3.80	1.17 (-0.20, 2.54)	0.09	0.35
BMI (kg/m²)	23.21 ± 2.58	23.70 ± 3.00	0.49 (-0.72, 1.70)	0.42	0.18

Abbreviations: *BMI* body mass index, *CI* confidence interval, *d* Cohen’s effect size

Elite athletes had lower body fat percentage (16.95 ± 4.45% vs. 18.42 ± 3.37%) and higher skeletal muscle mass percentage (45.29 ± 3.80% vs. 44.12 ± 3.01%) than amateur athletes. However, neither difference reached statistical significance (body fat, *p* = 0.06; skeletal muscle mass, *p* = 0.09). BMI was also comparable between groups (*p* = 0.42).

### Correlations between study variables

**Fig. 7:**
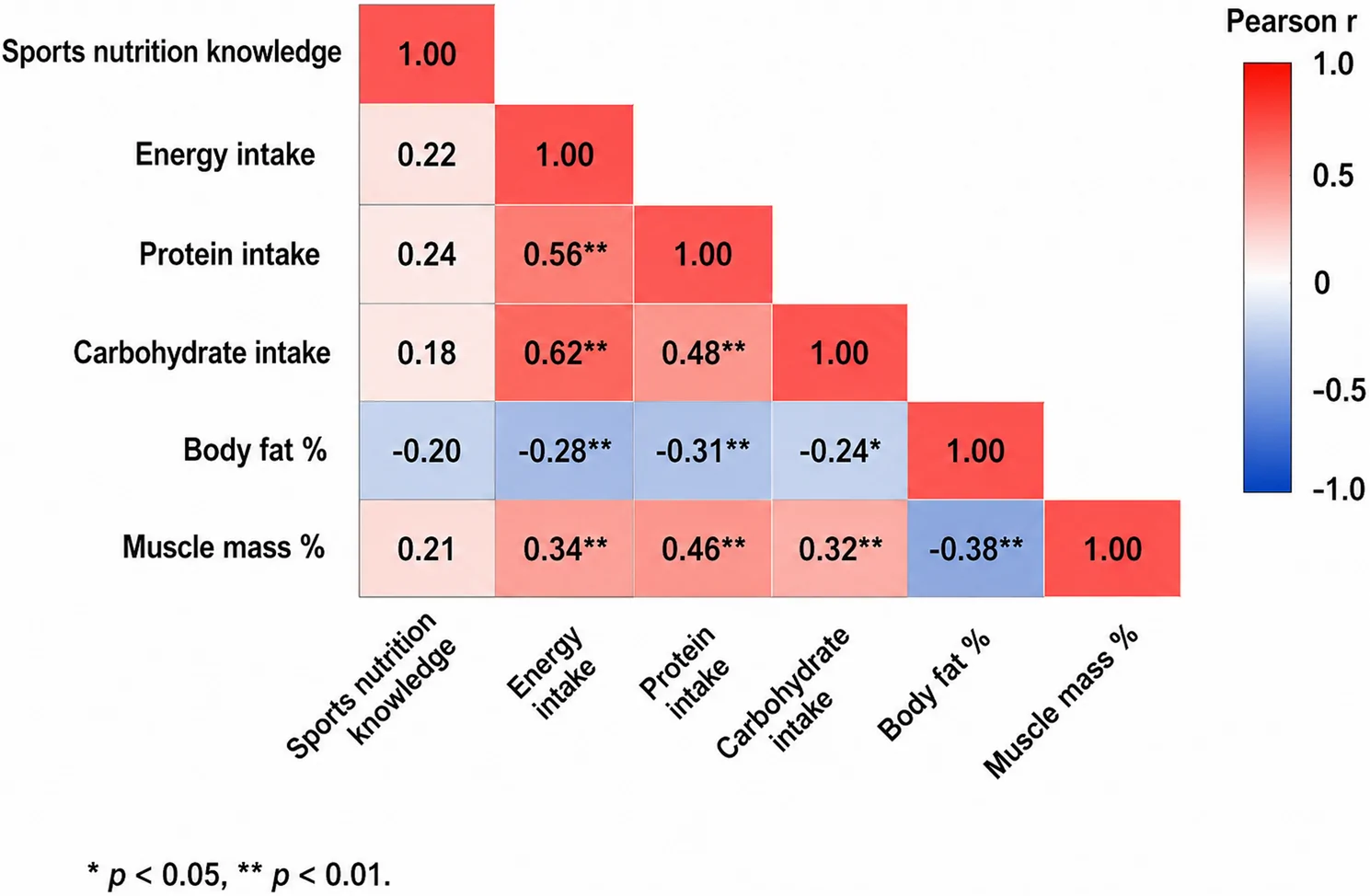
Pearson correlation matrix showing the relationships among sports nutrition knowledge, dietary intake variables, and body composition characteristics. Color intensity represents the magnitude and direction of Pearson correlation coefficients (r). *p < 0.05; **p < 0.01

Pearson correlation coefficients are presented in Table 4; Fig. 7.

**Table 4:** Correlations between sports nutrition knowledge, dietary intake, and body composition variables

Variable	Nutrition Knowledge	Energy Intake	Protein Intake	Carbohydrate Intake	Body Fat Percentage	Skeletal Muscle Mass Percentage
Nutrition knowledge	1.00	0.22	0.24	0.18	-0.20	0.21
Energy intake		1.00	0.56**	0.62**	-0.28**	0.34**
Protein intake			1.00	0.48**	-0.31**	0.46**
Carbohydrate intake				1.00	-0.24*	0.32**
Body fat percentage					1.00	-0.38**
Skeletal muscle mass percentage						1.00

Abbreviations: *r* Pearson correlation coefficient

*p < 0.05

** *p* < 0.01

Sports nutrition knowledge demonstrated weak correlations with the dietary intake and body composition variables examined.

Relative energy intake was positively correlated with protein intake (*r* = 0.56, *p* < 0.01) and carbohydrate intake (*r* = 0.62, *p* < 0.01). Protein intake was positively associated with skeletal muscle mass percentage (*r* = 0.46, *p* < 0.01) and negatively associated with body fat percentage (*r* = − 0.31, *p* < 0.01). Carbohydrate intake was positively associated with skeletal muscle mass percentage (*r* = 0.32, *p* < 0.01) and negatively associated with body fat percentage (*r* = − 0.24, *p* < 0.05). Body fat percentage was inversely correlated with skeletal muscle mass percentage (*r* = − 0.38, *p* < 0.01).

### Multiple regression models predicting body composition

Results of the multiple regression analyses are presented in Tables 5 and 6.

**Table 5:** Multiple linear regression model predicting body fat percentage

Predictor	Standardized β	*p*-value	VIF
Sports nutrition knowledge score	-0.12	0.18	1.21
Relative energy intake (kcal/kg/day)	-0.21	**0.02**	1.35
Protein intake (g/kg/day)	-0.29	**< 0.001**	1.42
Carbohydrate intake (g/kg/day)	-0.10	0.25	1.30
Elite competitive status	-0.24	**0.01**	1.18

Model statistics: *R²* = 0.42; Adjusted *R²* = 0.39; *F*(5,94) = 13.62; *p* < 0.001

Abbreviations: *β* standardized regression coefficient, *VIF* variance inflation factor, *R²* coefficient of determination

Bold values indicate statistically significant results (*p* < 0.05)

**Table 6:** Multiple linear regression model predicting skeletal muscle mass percentage

Predictor	Standardized β	*p*-value	VIF
Sports nutrition knowledge score	0.10	0.27	1.21
Relative energy intake (kcal/kg/day)	0.18	**0.04**	1.35
Protein intake (g/kg/day)	0.34	**< 0.001**	1.42
Carbohydrate intake (g/kg/day)	0.14	0.11	1.30
Elite competitive status	0.22	**0.02**	1.18

Model statistics: *R²* = 0.46; Adjusted *R²* = 0.43; *F*(5,94) = 16.01; *p* < 0.001

Abbreviations: β standardized regression coefficient, *VIF* variance inflation factor, *R²* coefficient of determination

Bold values indicate statistically significant results (*p* < 0.05)

The regression model for body fat percentage was statistically significant (R² = 0.42, adjusted R² = 0.39, *F*(5,94) = 13.62, *p* < 0.001). Protein intake (β = −0.29, *p* < 0.01), relative energy intake (β = −0.21, *p* = 0.02), and elite competitive status (β = −0.24, *p* = 0.01) were independently associated with lower body fat percentage. Sports nutrition knowledge and carbohydrate intake were not significantly associated with body fat percentage in the adjusted model.

The regression model for skeletal muscle mass percentage was also statistically significant (R² = 0.46, adjusted R² = 0.43, *F*(5,94) = 16.01, *p* < 0.001). Among the variables included in the adjusted model, protein intake showed the strongest independent association with skeletal muscle mass percentage (β = 0.34, *p* < 0.001), followed by elite competitive status (β = 0.22, *p* = 0.02) and relative energy intake (β = 0.18, *p* = 0.04).Sports nutrition knowledge and carbohydrate intake were not significantly associated with skeletal muscle mass percentage.

Regression diagnostics indicated no evidence of meaningful violations of model assumptions. Multicollinearity was not detected (all VIF < 5), and the assumptions of normality, linearity, homoscedasticity, and independence of residuals were satisfied. The Durbin–Watson statistics were 2.03 for the body fat percentage model and 1.96 for the skeletal muscle mass percentage model, indicating no evidence of residual autocorrelation.

## Discussion

### Overview of principal findings

This study examined the relationships among sports nutrition knowledge, dietary intake, and body composition in collegiate athletes while comparing amateur and elite competitors. Three principal findings emerged. First, elite athletes reported significantly higher energy, protein, and carbohydrate intake, together with greater meal frequency and daily fluid consumption, than amateur athletes. Second, sports nutrition knowledge scores were comparable between competitive levels despite these differences in dietary intake. Third, dietary intake variables, particularly protein intake, demonstrated stronger associations with body composition than sports nutrition knowledge. In the regression analyses, protein intake and competitive level were independently associated with body fat percentage and skeletal muscle mass, whereas sports nutrition knowledge was not.

By evaluating sports nutrition knowledge, dietary intake, and body composition within the same cohort, this study provides an integrated perspective on these interrelated factors in collegiate athletes. The findings are consistent with recent evidence indicating that dietary behaviors may be more closely associated with body composition than nutrition knowledge alone and emphasize the importance of examining nutritional knowledge together with actual dietary practices [21, 22].

### Sports nutrition knowledge versus dietary behavior

A notable finding of this study was that sports nutrition knowledge did not differ significantly between elite and amateur athletes, despite clear differences in dietary intake. Elite athletes reported higher energy, protein, and carbohydrate intake, whereas overall nutrition knowledge scores remained similar between groups. These findings suggest that differences in dietary behavior may occur even when athletes demonstrate comparable levels of sports nutrition knowledge.

This observation is consistent with previous research. Hopper et al. [23] reported that athletes frequently demonstrate moderate to good sports nutrition knowledge while still failing to meet recommended energy and macronutrient intakes. Similarly, Musau et al. [24] found only modest associations between nutrition knowledge and dietary behaviors, indicating that additional factors beyond knowledge may influence athletes’ food choices.

The present findings should be interpreted cautiously because this study did not directly assess factors such as access to nutrition professionals, previous nutrition education, food availability, or socioeconomic circumstances. Nevertheless, these variables have been identified in previous studies as potential influences on dietary behavior and may partly explain why athletes with similar nutrition knowledge demonstrate different dietary practices [10, 11].

The regression analyses further indicated that dietary intake variables, particularly protein intake, were independently associated with body composition, whereas sports nutrition knowledge was not. Similar findings have been reported by Devlin et al. [25], who observed stronger associations between dietary intake and body composition than between nutrition knowledge and body composition. Together, these findings suggest that nutrition knowledge alone may not adequately reflect athletes’ dietary behaviors or body composition. Collectively, these findings suggest that nutrition knowledge alone may not adequately reflect athletes’ dietary behaviors or body composition.

From a practical perspective, these findings support the value of combining nutrition education with individualized dietary assessment and behavioral support. Although the present cross-sectional study cannot determine whether such strategies improve dietary behaviors or body composition, previous intervention studies have reported favorable changes following individualized nutrition education and counseling [26, 27]. Accordingly, future longitudinal and intervention studies are warranted to determine whether improving the practical application of sports nutrition knowledge results in sustained improvements in dietary intake and body composition.

### Dietary intake according to competitive level

Elite athletes reported significantly higher energy, protein, and carbohydrate intake, consumed meals more frequently, and reported greater daily fluid intake than amateur athletes. These findings are consistent with previous studies showing that athletes competing at higher levels generally report dietary practices that more closely align with current sports nutrition recommendations [28].

Elite athletes also reported higher relative energy intake than amateur athletes. Current sports nutrition recommendations emphasize adequate energy availability to support training adaptation and recovery, whereas inadequate energy intake has been associated with impaired recovery, reduced training quality, and an increased risk of low energy availability [29–31]. Although energy intake was independently associated with body composition in the regression analyses, the cross-sectional design does not permit conclusions regarding the direction of this association.

Among the dietary variables examined, protein intake demonstrated the strongest association with body composition. Protein intake was independently associated with lower body fat percentage and higher skeletal muscle mass in the regression analyses, consistent with evidence supporting the role of adequate protein intake in muscle protein synthesis, recovery, and the maintenance of lean tissue during regular training [16]. Elite athletes also reported protein intakes that more closely approximated current sports nutrition recommendations than amateur athletes [28].

Elite athletes also reported higher carbohydrate intake than amateur athletes. Although carbohydrate intake was associated with body composition in the correlation analyses, it was not independently associated with body composition after adjustment for other variables in the regression models. This finding suggests that carbohydrate intake may be related to body composition in conjunction with other nutritional and training-related factors rather than as an isolated variable. Current guidelines continue to emphasize adequate carbohydrate availability to support glycogen restoration and repeated high-intensity exercise [32–34].

Similarly, elite athletes reported greater daily fluid intake than amateur athletes. Adequate hydration is recognized as an important component of sports nutrition because it supports thermoregulation, cardiovascular function, and exercise capacity, particularly during prolonged or high-intensity exercise [35]. However, hydration status was not measured directly in the present study, and therefore the higher reported fluid intake should not be interpreted as evidence of better hydration status.

Overall, competitive level was associated with differences in reported dietary intake. Because several potentially important factors, including training volume, nutrition support, and food availability, were not measured, these findings should be interpreted cautiously. Future longitudinal studies should examine these relationships while accounting for sport discipline, training characteristics, and the broader nutritional environment.

### Body composition and factors associated with body composition

Although elite athletes demonstrated lower body fat percentage and higher skeletal muscle mass than amateur athletes, these differences did not reach statistical significance. Nevertheless, the observed effect sizes suggest that these trends may be of practical interest. The absence of statistically significant between-group differences also indicates that competitive level alone may not adequately explain variation in body composition. Rather, body composition is likely associated with multiple interacting factors, including dietary intake, training characteristics, recovery practices, and individual physiological differences [36, 37].

The regression analyses provided additional insight into these relationships. Protein intake demonstrated the strongest independent association with lower body fat percentage and higher skeletal muscle mass, whereas sports nutrition knowledge was not independently associated with body composition after adjustment for dietary intake and competitive level. Similar findings have been reported by Devlin et al. [25], who observed stronger relationships between dietary intake and body composition than between nutrition knowledge and body composition. Collectively, these findings suggest that dietary intake variables may be more closely associated with body composition than nutrition knowledge alone.

Although relative energy intake and competitive level were also independently associated with body composition, these findings should be interpreted cautiously because the cross-sectional design does not permit conclusions regarding the direction of these associations. Approximately half of the variance in body composition remained unexplained, suggesting that additional factors not measured in this study may also contribute.

Previous studies have reported that dietary supplement use is common among competitive athletes, although supplementation practices are not always consistent with current evidence-based recommendations and frequently depend on individual nutritional requirements, sport-specific demands, and professional guidance [38–40]. Because dietary supplement use was not assessed in the present study, its potential contribution to dietary intake or body composition could not be evaluated and should therefore be considered in future research.

Several unmeasured training-related and contextual factors may also have been associated with the observed relationships. These include sport-specific training characteristics, nutrition support, and other environmental influences that were not assessed in the present study. Their potential contribution should therefore be considered when interpreting the findings and is discussed further in the study limitations.

Body composition was assessed using multi-frequency bioelectrical impedance analysis (BIA), a practical method widely used in athletic settings. Although BIA is influenced by factors such as hydration status and recent food intake, standardized assessment procedures were followed to minimize measurement error. Previous research has demonstrated acceptable reliability of modern multi-frequency BIA devices for monitoring body composition in athletic populations when standardized protocols are applied [41, 42]. Consequently, BIA represents a practical field-based assessment method, although it should not be considered a criterion measure of body composition.

Overall, the present findings indicate that body composition was associated with multiple nutritional, training-related, and contextual factors rather than a single characteristic. Future longitudinal and intervention studies should incorporate sport-specific analyses and comprehensive assessments of dietary intake, supplement use, training load, and nutrition support to further clarify these relationships.

### Strengths

This study has several strengths. First, it simultaneously assessed sports nutrition knowledge, dietary intake, and objectively measured body composition within the same cohort of collegiate athletes, providing a comprehensive evaluation of the relationships among these variables [22, 25]. Second, dietary intake was collected using standardized dietary records, and body composition was assessed using multi-frequency bioelectrical impedance analysis under standardized testing conditions, supporting the consistency of the measurements despite the recognized limitations of BIA as a field-based method [41, 42]. Third, the inclusion of both amateur and elite athletes enabled comparisons across competitive levels, providing additional insight into nutritional characteristics within a collegiate athletic population. Although participants represented multiple sports, classifying athletes according to predefined competitive-level criteria allowed standardized comparisons between groups.

### Limitations

Several limitations should be considered when interpreting the findings of this study. First, the cross-sectional design precludes conclusions regarding the direction or causality of the observed associations among sports nutrition knowledge, dietary intake, and body composition.

Second, participants represented multiple sport disciplines with different physiological demands, nutritional requirements, and training characteristics. Because analyses were not stratified by sport, discipline-specific differences may have influenced the observed associations. Furthermore, although athletes were classified using objective operational criteria based on competitive experience and supervised training frequency, these criteria were developed specifically for this study and may not correspond directly to athlete classification systems used in other sports or research settings. Consequently, comparisons with studies using alternative classification frameworks should be made with caution.

Third, important training-related variables, including total training volume, training intensity, seasonal training load, and resistance versus endurance training exposure, were not quantified. These factors are known to be associated with dietary requirements and body composition and may therefore have acted as confounding variables.

In addition, several contextual factors that may influence dietary behaviors were not assessed, including previous sports nutrition education, access to sports dietitians or nutrition support services, socioeconomic status, living arrangements, food availability, and nutrition-related support provided by coaches or athletic programs. Consequently, some of the differences observed between amateur and elite athletes may reflect variation in these contextual factors rather than competitive level itself.

Finally, dietary intake was assessed using self-reported food records, which are susceptible to recall bias and underreporting despite the use of standardized collection procedures. Body composition was measured using multi-frequency bioelectrical impedance analysis (BIA), a practical and reliable field-based method for athlete monitoring, although it is not considered a criterion reference technique.

Future longitudinal and intervention studies should incorporate sport-specific analyses, objective measures of training load and energy expenditure, and comprehensive assessments of nutrition support and other contextual influences to further clarify the relationships among sports nutrition knowledge, dietary intake, and body composition in collegiate athletes.

### Practical applications

The findings have practical implications for coaches, sports dietitians, and university athletic programs. Routine nutritional assessment should include evaluation of both dietary intake and sports nutrition knowledge to provide a more comprehensive understanding of athletes’ nutritional status. Particular attention should be given to achieving adequate energy, protein, and carbohydrate intake and maintaining appropriate hydration practices in accordance with current sports nutrition guidelines [26, 27, 35].

Because this study did not assess nutrition support services, food availability, or other contextual factors, no conclusions can be drawn regarding their contribution to the observed differences between competitive levels. Nevertheless, individualized dietary assessment and evidence-informed nutrition guidance may help identify nutritional gaps and support athletes in meeting current sports nutrition recommendations. Future intervention studies are needed to determine the effectiveness of these strategies to evaluate whether these strategies improve dietary behaviors, body composition, and performance-related outcomes.

## Conclusion

This study examined the relationships among sports nutrition knowledge, dietary intake, and body composition in collegiate athletes while comparing amateur and elite competitors. Elite athletes reported higher energy, protein, and carbohydrate intake, greater meal frequency, and higher daily fluid intake than amateur athletes, whereas sports nutrition knowledge scores were comparable between groups. Dietary intake variables, particularly protein intake, were more strongly associated with body composition than sports nutrition knowledge, and protein intake remained independently associated with body fat percentage and skeletal muscle mass in the regression analyses.

Overall, the findings suggest that dietary intake may be more closely associated with body composition than sports nutrition knowledge alone in this cohort of collegiate athletes. However, because of the cross-sectional design and the potential influence of unmeasured confounding variables, these associations should not be interpreted as evidence of causal relationships. Rather, body composition is likely to be associated with multiple interacting factors, including dietary intake, training characteristics, sport-specific demands, and the broader nutritional support environment.

From a practical perspective, the findings support the value of assessing dietary behaviors alongside sports nutrition knowledge when evaluating athletes’ nutritional status. Future prospective and intervention studies are warranted to evaluate whether improvements in dietary practices, nutrition support, and sport-specific nutritional strategies are associated with favorable changes in body composition and athletic performance while accounting for potential confounding factors.

## Supplementary Information

Supplementary Material 1.

## Data Availability

All raw data underlying the findings of this study are available from the corresponding author upon reasonable request and without undue restriction.
