# Supplementary material for: Competitive level is associated with dietary practices but not sports nutrition knowledge in collegiate athletes: associations with body composition profiles
Source: BMC Sports Sci Med Rehabil. 2026 Aug 20;18:368. doi: 10.1186/s13102-026-01974-5 (PMC13491743; doi:10.1186/s13102-026-01974-5)
Supplement: Supplementary file 1 [file 13102_2026_1974_MOESM1_ESM.docx]

STROBE Statement—checklist of items that should be included in reports of observational studies

|  | Item No. | Recommendation | Page  No. | Relevant text from manuscript |
| --- | --- | --- | --- | --- |
| **Title and abstract** | 1 | (*a*) Indicate the study’s design with a commonly used term in the title or the abstract | 1 | The study design (cross-sectional study) was clearly indicated in the title. |
|  |  | (*b*) Provide in the abstract an informative and balanced summary of what was done and what was found | 1-2 | The abstract provides a balanced summary of the study background, objectives, methods, results, and conclusions. |
| Introduction | | | |  |
| Background/rationale | 2 | Explain the scientific background and rationale for the investigation being reported | 2-5 | The scientific background and rationale for investigating relationships among sports nutrition knowledge, dietary intake, and body composition in collegiate athletes were described in the Introduction |
| Objectives | 3 | State specific objectives, including any prespecified hypotheses | 5 | The study objectives and hypotheses were clearly stated at the end of the Introduction. |
| Methods | | | |  |
| Study design | 4 | Present key elements of study design early in the paper | 5 | A cross-sectional observational study design was employed and described in the Methods section. |
| Setting | 5 | Describe the setting, locations, and relevant dates, including periods of recruitment, exposure, follow-up, and data collection | 5-6 | The study setting, participant recruitment procedures, and data collection period were described in the Methods section |
| Participants | 6 | (*a*) *Cohort study*—Give the eligibility criteria, and the sources and methods of selection of participants. Describe methods of follow-up  *Case-control study*—Give the eligibility criteria, and the sources and methods of case ascertainment and control selection. Give the rationale for the choice of cases and controls  *Cross-sectional study*—Give the eligibility criteria, and the sources and methods of selection of participants | 5- 6 | Eligibility criteria, recruitment procedures, and participant characteristics were described in the Participants section. |
|  |  | (*b*) *Cohort study*—For matched studies, give matching criteria and number of exposed and unexposed  *Case-control study*—For matched studies, give matching criteria and the number of controls per case |  |  |
| Variables | 7 | Clearly define all outcomes, exposures, predictors, potential confounders, and effect modifiers. Give diagnostic criteria, if applicable | 7-9 | Sports nutrition knowledge, dietary intake variables, body composition characteristics, and competitive level were clearly defined as study variables. |
| Data sources/ measurement | 8* | For each variable of interest, give sources of data and details of methods of assessment (measurement). Describe comparability of assessment methods if there is more than one group | *8-9* | *Sports nutrition knowledge was assessed using the Nutrition for Sport Knowledge Questionnaire (NSKQ), dietary intake was assessed using 3-day dietary records, and body composition was measured using the InBody 770 multifrequency bioelectrical impedance analyzer.* |
| Bias | 9 | Describe any efforts to address potential sources of bias | 21 | Potential sources of bias, including self-reported dietary intake, single-institution recruitment, and the use of bioelectrical impedance analysis rather than criterion body-composition assessment methods, were acknowledged and discussed in the Limitations section. |
| Study size | 10 | Explain how the study size was arrived at | 6-7 | Sample size determination and participant recruitment procedures were described in the Participants section. |

| Quantitative variables | 11 | Explain how quantitative variables were handled in the analyses. If applicable, describe which groupings were chosen and why | 7-10 | Continuous variables, including nutrition knowledge scores, dietary intake variables, and body composition parameters, were analyzed as quantitative measures and reported as mean ± standard deviation. |
| --- | --- | --- | --- | --- |
| Statistical methods | 12 | (*a*) Describe all statistical methods, including those used to control for confounding | 10-11 | independent-samples t-tests, Pearson correlation analyses, and multiple linear regression analyses were performed using IBM SPSS Statistics version 29.0. Effect sizes (Cohen’s d), 95% confidence intervals, and regression diagnostics were reported. |
|  |  | (*b*) Describe any methods used to examine subgroups and interactions | 10-11 | Comparisons were conducted according to competitive level (elite versus amateur athletes). |
|  |  | (*c*) Explain how missing data were addressed | 11 | No missing data were identified; therefore, no imputation procedures were required. |
|  |  | (*d*) *Cohort study*—If applicable, explain how loss to follow-up was addressed  *Case-control study*—If applicable, explain how matching of cases and controls was addressed  *Cross-sectional study*—If applicable, describe analytical methods taking account of sampling strategy |  | Not applicable. |
|  |  | (*e*) Describe any sensitivity analyses |  | Not applicable. |
| Results | | | | |
| Participants | 13* | (a) Report numbers of individuals at each stage of study—eg numbers potentially eligible, examined for eligibility, confirmed eligible, included in the study, completing follow-up, and analysed | 11 | The numbers of eligible, recruited, and analyzed participants were reported in the Results section. |
|  |  | (b) Give reasons for non-participation at each stage | 6-7 | Reasons for participant exclusion were reported where applicable |
|  |  | (c) Consider use of a flow diagram |  | "Not applicable; all eligible participants completed the study procedures." |
| Descriptive data | 14* | (a) Give characteristics of study participants (eg demographic, clinical, social) and information on exposures and potential confounders | 11-12 | Participant demographic characteristics, nutrition knowledge scores, dietary intake variables, and body composition measures were reported in Tables 1–3. |
|  |  | (b) Indicate number of participants with missing data for each variable of interest | 11 | No missing data were identified for the analyzed variables. |
|  |  | (c) *Cohort study*—Summarise follow-up time (eg, average and total amount) |  | Not applicable. |
| Outcome data | 15* | *Cohort study*—Report numbers of outcome events or summary measures over time | *12-15* | *Nutrition knowledge scores, dietary intake variables, and body composition outcomes were reported for both elite and amateur athlete groups.* |
|  |  | *Case-control study—*Report numbers in each exposure category, or summary measures of exposure |  |  |
|  |  | *Cross-sectional study—*Report numbers of outcome events or summary measures |  |  |
| Main results | 16 | (*a*) Give unadjusted estimates and, if applicable, confounder-adjusted estimates and their precision (eg, 95% confidence interval). Make clear which confounders were adjusted for and why they were included | 12-16 | Between-group comparisons were reported using p-values, 95% confidence intervals, and Cohen’s d effect sizes. Multiple linear regression models were used to identify independent predictors of body-fat percentage and muscle-mass percentage. |
|  |  | (*b*) Report category boundaries when continuous variables were categorized |  | Not applicable |
|  |  | (*c*) If relevant, consider translating estimates of relative risk into absolute risk for a meaningful time period |  | Not applicable |

| Other analyses | 17 | Report other analyses done—eg analyses of subgroups and interactions, and sensitivity analyses | 15-16 | Pearson correlation analyses and multiple linear regression models were conducted to examine relationships among sports nutrition knowledge, dietary intake, and body composition variables |
| --- | --- | --- | --- | --- |
| Discussion | | | | |
| Key results | 18 | Summarise key results with reference to study objectives | 16-17 | Elite athletes reported significantly higher energy, protein, and carbohydrate intake than amateur athletes despite similar nutrition knowledge scores. Protein intake demonstrated the strongest association with muscle-mass percentage. |
| Limitations | 19 | Discuss limitations of the study, taking into account sources of potential bias or imprecision. Discuss both direction and magnitude of any potential bias | 20-21 | The limitations of the study, including the cross-sectional design, self-reported dietary assessment, male-only sample, use of BIA rather than criterion body-composition methods, and pooling of athletes from multiple sports, were discussed. |
| Interpretation | 20 | Give a cautious overall interpretation of results considering objectives, limitations, multiplicity of analyses, results from similar studies, and other relevant evidence | 16-20 | The findings were interpreted in relation to previous literature, study limitations, and the distinction between nutrition knowledge and practical dietary implementation. |
| Generalisability | 21 | Discuss the generalisability (external validity) of the study results | 20-21 | the potential generalizability of the findings to other athlete populations was discussed, together with factors limiting external validity. |
| Other information | |  | | |
| Funding | 22 | Give the source of funding and the role of the funders for the present study and, if applicable, for the original study on which the present article is based | 23 | Sources of funding and the role of the funders were reported in the Funding section. |

*Give information separately for cases and controls in case-control studies and, if applicable, for exposed and unexposed groups in cohort and cross-sectional studies.

**Note:** An Explanation and Elaboration article discusses each checklist item and gives methodological background and published examples of transparent reporting. The STROBE checklist is best used in conjunction with this article (freely available on the Web sites of PLoS Medicine at http://www.plosmedicine.org/, Annals of Internal Medicine at http://www.annals.org/, and Epidemiology at http://www.epidem.com/). Information on the STROBE Initiative is available at www.strobe-statement.org.
